# Cathepsins in digestive cancers

**DOI:** 10.18632/oncotarget.16677

**Published:** 2017-03-29

**Authors:** Siyuan Chen, Hui Dong, Shiming Yang, Hong Guo

**Affiliations:** ^1^ Department of Gastroenterology, Xinqiao Hospital, Third Military Medical University, Chongqing, China

**Keywords:** cathepsin, digestive cancer, proliferation, migration, invasion

## Abstract

Cathepsins are lysosomal peptidases belonging to the papain family, and based on their catalytic sites, these enzymes can be divided into serine, cysteine and aspartic proteases. The studies conducted to date have identified, 15 types of cathepsins that are widely distributed in intracellular and extracellular spaces. These proteases participate in various pathological activities, including the occurrence and development of human cancers. Several recent studies suggest that cathepsins, particularly cathepsins B, D, E and L, contribute to digestive tumorigenesis. Cathepsins were found to promote the development of most digestive cancers except liver cancer, in which they might have the opposite effects. Due to their important roles in digestive tumors, cathepsins might be therapeutic targets for the treatment of digestive cancers.

## INTRODUCTION

Cathepsins are lysosomal globular proteases belonging to the papain family [1]. These enzymes are widely distributed in intracellular and extracellular spaces. Cathepsins were first proposed in the 1920s. Since the crystal structure of the first cathepsin (cathepsin B) was determined, researchers in this field have successively identified an increasing number of cathepsins and their inhibitors. To date, more than 20 types of cathepsins, ranging from cathepsin A to Z, have been reported in various organisms, including animals, plants and microorganisms. Fifteen types of cathepsins are expressed in humans, and these are divided into serine, cysteine and aspartic proteases [1]. This protease family plays important roles in many biological activities, such as the growth and development of organisms, immune responses, and the development of various diseases, including tumorigenesis [2–7].The relationship between cathepsins and tumors has been studied for approximately 40 years [8]. Various cathepsins contribute to extracellular matrix (ECM) degradation and remodeling in the tumor microenvironment [9, 10], resulting in the acceleration of tumor progression and invasion [11–16]. In general, a higher level of cathepsins is associated with a poorer prognosis [17–19]. Over the past few years, cathepsins were found to be overexpressed in digestive cancer cells and to particularly promote cancer invasion and metastasis. Here, we review the relationship between human cathepsins and digestive cancers.

## CLASSIFICATION OF CATHEPSINS

Fifteen types of human cathepsins have been reported to date. Based on their catalytic mechanism, cathepsins are subdivided into serine (A and G), cysteine (B, C, F, H, K, L, O, S, V, W and X) and aspartic proteases (D and E) [1, 20]. We describe the classification of cathepsins in Table 1.

**Table 1:** Human cathepsin isoforms

Species	Protein family	Number of amino acids (aa)	EC No.	Location
**Cathepsin A**		480	3.4.16.5	Brain, skin, placenta, platelet, liver
**Cathepsin G**		225	3.4.21.2	Skin, monocytes, neutrophils
**Cathepsin D**		412	3.4.23.5	Spleen, kidney, liver, melanoma, plasma, platelets
**Cathepsin E**		401	3.4.23.34	Brain, intestine, stomach, Erythrocytes, lymph nodes, skin, spleen, lung
**Cathepsin B**		339	3.4.22.1	Liver, kidney, thyroid gland, spleen
**Cathepsin C**		463	3.4.14.1	Lung, spleen, kidney, placenta, cytotoxic T lymphocytes
**Cathepsin F**		484	3.4.22.41	Brain, heart, skeletal muscle, testis, ovary, macrophages
**Cathepsin H**		335	3.4.22.16	Liver, kidney, spleen
**Cathepsin K**		329	3.4.22.38	Osteoclasts, macrophages, epithelial cells of the gastrointestinal, respiratory and urinary tracts in human embryos and fetuses, lung
**Cathepsin L**		333	3.4.22.15	Liver, thyroid gland, kidney
**Cathepsin O**		321	3.4.22.42	Liver, kidney, placenta, ovary
**Cathepsin S**		331	3.4.22.27	Spleen, lymph nodes, antigen-presenting cells, heart
**Cathepsin V**		334	3.4.22.43	Cornea, testes, thymus
**Cathepsin W**		376	3.4.22.-	Spleen, lymph nodes (specifically cytotoxic T lymphocytes)
**Cathepsin X**		303	3.4.18.1	Liver, kidney, placenta, lung

## SYNTHESIS AND MODIFICATION OF CATHEPSINS

**Figure 1:**
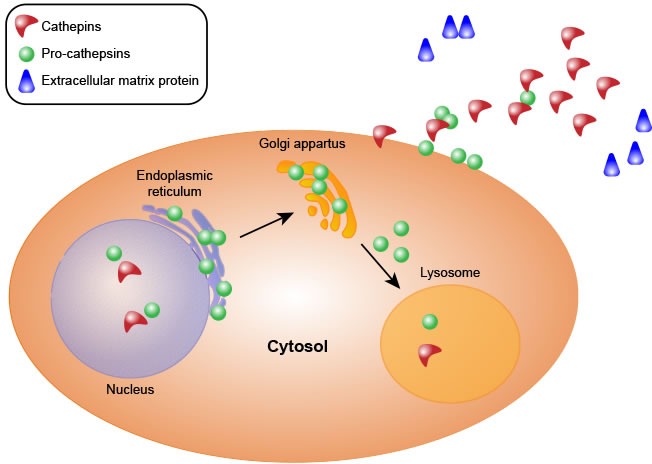
Cathepsin synthesis

Almost all types of cathepsins share a common synthetic pathway. First, cathepsins are synthesized as precursors without activity in the ribosome. These precursors are composed of signal peptides, precursor peptides and catalytic domains. Second, the precursors are translocated to the endoplasmic reticulum, where the signal peptide of each procathepsin is hydrolyzed, yielding the precursor peptide and the catalytic domain, and the protein is also glycosylated in the endoplasmic reticulum. Third, the proteins are transported to the Golgi apparatus, where they are further glycosylated and phosphorylated to form mannose-6-phosphate proteins. Finally, the modified proteins are recognized by mannose-6-phosphate receptors in the lysosome, where the proenzymes are hydrolyzed at low pH, resulting in the removal of the prodomains to yield active and mature cathepsins [6, 21–24] (Figure 1).

However, recent studies showed that the mannose-6-phosphate marker is not always necessary. Sortilin, a multifunctional glycoprotein that serves as a multi-ligand receptor, can induce protein translocation. Specifically, sortilin can bind to cathepsins D and H in the Golgi apparatus and mediates their transport from the Golgi to the lysosome [25].

Procathepsins are activated *via* two proteolytic pathways [1, 26]. The first is autocatalytic activation [27–30]. Glycosaminoglycans (GAGs) and negatively charged surfaces have been reported to facilitate the autocatalytic activation of cysteine cathepsins. The binding of GAGs induces a conformational change in the cathepsin zymogen and loosens the interaction between the propeptide and the mature part of the enzyme, enabling easier processing by another procathepsin molecule. Notably, although GAGs play important roles in cathepsin activation, the GAG-binding surface has not been demonstrated to be shared by all cathepsins [31–33]. Another activation method is catalysis by other proteases. For example, cathepsins C and X are activated by cathepsin L or S to eliminate the prodomains, and cathepsin B is cleaved and activated by cathepsin D [34, 35].

## CATHEPSIN LOCALIZATION

Cathepsins are mainly located in the lysosome and prefer an acidic environment, but their location can also change under different conditions. These enzymes might be released into the nucleus to shear histone and regulate gene expression [36] or be transported to the cell surface and secreted into the ECM to perform different functions [31, 37]. For example, in tumor cells, cathepsins translocate to the cell surface to degrade the ECM, allowing the invasion or metastasis of tumor cells. Some types of cathepsins (such as cathepsin B and L) can also migrate into the circulatory system and are detected in serum, but their expression level can present substantial changes, making them potential clinical indices.

## CATHEPSIN FUNCTIONS

The original meaning of cathepsin is “digest”, and the basic function of proteases is hydrolysis. Over the past few years, researchers have demonstrated that the cathepsin family participates in various physiological and pathological processes. These enzymes participate in development and differentiation [21], such as angiogenesis [38], the hair follicle cycle [39] and the occurrence of sperm and ovum during reproduction [40]. Moreover, cathepsins are involved in organism apoptosis [41], immune responses [5, 42] and skeletal metabolism [43]. In addition, the processing of some hormones depends on cathepsins [21]. For instance, thyroid hormones must be processed by cathepsin B for maturation [44]. Cathepsins also contribute to some diseases, such as osteoporosis, osteoarthritis, pycnodysostosis, rheumatoid arthritis, Down syndrome, Alzheimer's disease and asthenic bulbar paralysis [7, 40, 45, 46].

## CATHEPSIN INHIBITORS

Since the last century, when the first cathepsin crystal structure was determined, cathepsin inhibitors have been studied in increasing detail. Cathepsin inhibitors combine with some groups in the active center of cathepsins to diminish or block cathepsin activity, but these compounds do not denature the enzymes themselves. Based on their binding properties, cathepsin inhibitors include reversible and irreversible inhibitors. Depending on the source, inhibitors are divided into endogenous inhibitors and synthetic inhibitors. Endogenous inhibitors include thyropins, the precursor peptide, the serpin family, the cystatin family, α2-macroglobulin and cytotoxic T lymphocyte antigen-2β [5, 6, 47–49]. Compared with endogenous inhibitors, there are more synthetic inhibitors, such as aldehydes, ketones [47, 48], nitriles [50], epoxysuccinyls [47, 48], hydrazones, carbohydrazides [47, 51], vinyl sulfones [52], β-lactams [53] and phosphoryl dipeptides [54] (shown in Table 2). In general, synthetic inhibitors have been developed more rapidly than endogenous inhibitors because they are easier to prepare and modify; thus, synthetic inhibitors might be more specific for cathepsins. More accurate and effective inhibitors have been designed, and these inhibitors have become increasingly more useful, with some inhibitors having entered clinical trials. We believe that the development of related instruments and an in-depth analysis of cathepsin structures will advance the field of inhibitor research.

**Table 2:** Cathepsin inhibitors

Inhibitor source	Inhibitor
Endogenous inhibitors	Thyropins
	Precursor peptide
	Serpin family
	Cystatin family
	α2-Macroglobulin
	Cytotoxic T lymphocyte antigen-2β
Synthetic inhibitors	Aldehydes
	Ketones
	Nitriles
	Epoxysuccinyls
	Hydrazones
	Carbohydrazides
	Vinyl Sulfones
	β-lactams
	Phosphoryl dipeptides

## CATHEPSINS AND DIGESTIVE CANCERS

Digestive cancers include esophageal cancer, gastric cancer, colorectal cancer, pancreatic cancer and liver cancer. Similar to other cancers, digestive cancers exhibit a complex progression *via* multistep pathways involving the activation of oncogenes, such as K-sam and c-met [55], and the inactivation of anti-oncogenes, such as adenomatous polyposis coli (APC) [56] and tumor protein 53 (TP53) [57]. Many cathepsins, as tumor-promoting factors, contribute to digestive cancer development. Their expression is up-regulated in various digestive cancers, and the enzymes are activated and translocated during the progression of tumor development.

Digestive cancers progress through several important stages, and cathepsins take part in the relevant processes. Cathepsins contribute to at least three stages of cancers. First, a major feature of cancer cells is their enormous proliferative capacity, which requires the participation of various cytokines, and cathepsins can process these into mature proteins to promote cell division. Second, the growth of cancer cells requires blood vessels to provide nutrients and exclude metabolites. In this stage, cathepsins contribute to degradation of the vascular basement membrane and activate growth factors to promote angiogenesis. In addition, when secreted into pericellular environments, cathepsins cleave laminin, collagen, elastin, E-cadherin, and other matrix proteins [14], degrading the junctions between cells and the ECM and thereby allowing cancer cells to invade or metastasize [20].

As shown in a previous study, even though the cathepsin family is very important in digestive cancers, not all of its members are required for cancer development. Of all the cathepsins, cathepsins B and L are the two most frequently researched cathepsins in digestive tumors. Many reports on cathepsins D and E are available in the literature, but fewer studies have examined the other cathepsins. Interestingly, cathepsins function as a network and can substitute for each other when necessary. As shown by Akkari et al. [41], cathepsins have a compensatory mechanism in cancers. For instance, cathepsins B and S are predominantly responsible for pancreatic neuroendocrine tumor growth and invasion. The deletion of cathepsins B and S in RIP1-Tag2 mice yielded no differences in tumor invasion. Researchers have detected a significant increase in the levels of cathepsin Z, which is expressed at lower levels under normal conditions [41], indicating that other cathepsins might compensate for a loss of the main type of cathepsin. However, the mechanism underlying the activation of the compensatory pathway remains unknown and is worth further exploration.

## ESOPHAGEAL CANCER

Esophageal cancer is the sixth leading cause of cancer-related death. Once this type of cancer develops, it will rapidly spread and can invade other tissues [58]. Cathepsins B, C, D, K, and S are up-regulated in esophageal adenocarcinoma [59].

Cathepsin B is a potential target for detecting esophageal cancer [60] because it is expressed at low levels in the normal esophageal mucosa but is significantly overexpressed in esophageal cancer [61]. Habibollahi et al. used white-light upper endoscopy combined with near-infrared imaging to detect cathepsin B and thereby screen for esophageal adenocarcinoma [62]. The results indicate that cathepsin B appears to have huge potential for clinical applications.

As shown by Andl et al., the loss of tumor suppressor genes, such as E-cadherin and transforming growth factor type II receptor, initiates esophageal cell invasion, and this effect is further promoted by cathepsin B, resulting in increased levels of transforming growth factor-β (TGFβ) and thereby aiding the development of cancer [63].

Moreover, cathepsin D contributes to the development of esophageal cancer [64]. Previous studies have demonstrated that cathepsin D exacerbates the invasion of esophageal squamous cell carcinoma, and high cathepsin D expression is associated with poor prognosis [65].

## GASTRIC CANCER

Gastric cancer is the fourth most common cancer, and has one of the highest incidences of malignant cancer. Several types of cathepsins have functions in gastric cancers.

Cathepsin B, which is increasing up-regulated during tumor stage progression (T, N and TNM stages), is associated with poor prognosis in patients and an increase in tumor size. Additionally, a high cathepsin B expression level decreases laminin expression to promote gastric cancer cell invasion and metastasis [66].

In early gastric cancer, cathepsin D activates micro-lymph node metastasis [67], and studies have shown that human telomerase reverse transcriptase (hTERT) can stimulate cathepsin D expression by activating early growth response protein 1 (an important nuclear transcription factor that can promote cell proliferation), thereby inducing cancer cell invasion [68]. Anterior gradient 2 (AGR2), a p53 suppressor that is widely expressed in many tumors, is known to stimulate the proliferation and development of cancer cells. The expression of cathepsins B and D is post-transcriptionally induced by AGR2 to promote cancer cell dissemination [69]. Moreover, the oncogene c-myc is involved in the positive regulation of cathepsins. In addition to external factors, cathepsins can also activate each other. It has been reported that cathepsin D activates cathepsin B and then activates urokinase-type plasminogen activator to increase the number of malignant cancer cells [70–72].

Cathepsin E also presents a high expression level in gastric carcinoma and signet-ring cell carcinomas of the stomach [73]. Decreasing the expression of cathepsin E will reduce the differentiation of gastric tumors [74, 75]. Because cathepsin E has the ability to promote differentiation, it might serve as a marker of gastric differentiation [75].

Coronin 3 is an F-actin-binding and F-actin-interacting protein that is involved in the regulation of actin-dependent biological processes, such as cell motility and migration. Recent research showed that coronin 3 is involved in the development and metastasis of a variety of malignant tumors. Cathepsin K was shown to be positively correlated with coronin 3, which is activated during metastasis of the MKN28-M gastric cell line [76].

Forkhead box O3A has been shown to increase cathepsin L promoter activity, leading to an increase in cathepsin L expression and facilitating gastric cancer cell migration and invasion. Moreover, cathepsin L overexpression represses the expression of E-cadherin, causing gastric cancer cells to undergo the epithelial-mesenchymal transition [77]. In addition, cathepsin L also participates in the venous invasion of tumors [78].

Cathepsin S is up-regulated in 16 gastric cell lines and more than a thousand clinical samples. It can influence the expression levels of 197 proteins, one-third of which participate in cellular movement. The migration and invasion of gastric cancer cells is suppressed by the knockdown of cathepsin S. A clinical study showed that the serum cathepsin S level in early stages is lower than that at later stages and that patients with a high serum cathepsin S level have a poorer survival rate. Cathepsin S is positive correlated with gastric cancer development and was recently proposed as a new biomarker for the diagnosis and prognosis of gastric cancer [79].

The cathepsin X level is increased in *H. pylori*-infected gastric mucosa and gastric cancer [80]. Cathepsin X up-regulation is related to the tumorigenesis of gastric cancer and is directly associated with higher invasiveness *in vitro*. Ribosomal phosphoprotein P0 (RPLP0) can mediate protein synthesis, gene transcription and DNA modification, and cathepsin X interacts with RPLP0 in the N87 gastric cell line to promote cell cycle and resistance to apoptosis. The knockdown of cathepsin X inhibits the proliferation of gastric cancer cells [81].

As mentioned above, cystatin is an inhibitor that forms very tight equimolar complexes with cysteine proteases, competing with substrates to block the activities of these proteases. Reductions in cathepsin activity (cathepsins B, L, and V) by cystatin in gastric cancer might be useful for preventing gastric tumorigenesis [82]. In addition, some drugs have been developed based on the characteristics of cathepsins. For example, based on the overexpression of cathepsin B in tumors, a prodrug of doxorubicin (Ac-Phe-Lys-PABC-ADM) was designed. Cathepsin B cleaves the Phe-Lys dipeptide at the Lys-PABC bond, releasing doxorubicin to kill gastric cells and reducing the toxicity of doxorubicin [83]. These cathepsin-based antitumor drugs might become a new method of tumor treatment.

## COLORECTAL CANCER

Colon carcinoma is a major cause of death, particularly in the western world [84]. In general, cathepsins B, D, L and H are significantly up-regulated in colorectal carcinoma but are expressed at low or undetectable levels in normal tissue, suggesting that these proteins are involved in colorectal carcinoma growth and development.

Chan et al. tested 558 participants with colon cancer and found that 82% were positive for cathepsin B [85]. Cavallo-Medved et al. validated these results and revealed that up-regulated levels of cathepsin B are associated with poor prognosis [86]. The localization of cathepsin B is likely to change during tumorigenesis. In the normal colorectal mucosa, cathepsin B is likely located in the epithelium and is only active in the older cells at the colorectal surface. In contrast, in tumor tissues, cathepsin B is localized at the base of cells close to the basement membrane. Moreover, the location of cathepsin B is unchanged in most well-differentiated and half of moderately differentiated colon carcinomas, whereas this enzyme is diffusely spread throughout the cytoplasm in poorly differentiated colon carcinomas [84].

Compared with cathepsin B-negative tumors, cathepsin B-positive colon cancers are more likely to have K-ras and BRAF mutations and have higher multivariate hazard ratios [85]. A previous study investigated two colon carcinoma cell lines, namely HCT-116 (with a mutated K-ras allele) and HKh-2 (with a disruption in the mutated allele), and showed that cathepsin B expression and activity are higher in HCT116 cells than in HKh-2 cells. The researchers speculated that active K-ras increases cathepsin B, and cathepsin B then initiates a proteolytic cascade in colon carcinoma cells [86]. In addition to K-ras, other regulators of cathepsin B have also been identified. Using multi-cellular tumor spheroid cocultures of colon cancer cells to reveal the mechanism, researchers have shown that cathepsin B up-regulation is mediated by the mitogen-activated protein kinase and p38 signaling pathways.

In the early stages of colorectal cancer, cathepsin X is up-regulated and functions to stabilize tumor cell formation. Interestingly, its function changes as the tumor progresses, and loss of cathepsin X contributes to tumor progression and local invasion [87]. The findings suggest that the functions of cathepsins in tumors are complex and might differ depending on the stage of cancer development.

## PANCREATIC CANCER

Pancreatic cancer is the fifth leading cause of malignancy-related death, exhibits systemic micrometastasis and has a poorer prognosis than other human cancers. The overall five-year survival rate among patients with pancreatic cancer is less than 5% [88]. Cathepsins show very moderate reactivity in normal pancreatic tissue but are up-regulated in pancreatic cancer cells. The study by Niedergethmann et al. showed that cathepsins B and L contribute to perineural invasion [89]. Singh et al. performed a study of 127 patients with pancreatic cancer and found that their plasma cathepsin L levels were elevated and are associated with a poor prognosis [90]. The available evidence shows that cathepsin L is overexpressed following myc activation in the β-cell compartment and might be a target of myc-driven tumorigenesis [91].

Moreover, cathepsin E is a candidate marker for the diagnosis of pancreatic cancer. Cathepsin E is overexpressed in pancreatic intraepithelial neoplasia and pancreatic ductal adenocarcinoma (PDAC) [92]. Confocal laser endomicroscopy has been used *in vivo* for detecting the cathepsin E levels and monitoring pancreatic carcinogenesis [93]. It has been demonstrated that cathepsin E exhibits greater sensitivity, specificity and diagnostic accuracy compared with the CA19-9, carcinoembryonic antigen and K-ras mutations [94].

Studies have been performed to determine the mechanism of cathepsin in pancreatic cancer. Hedgehog (Hh) signaling is involved in pancreatic cancer development and can regulate invasion by increasing cathepsin B expression. Hwang et al. [95] treated the PANC-1 cell line with cyclopamine (Hh signal inhibitor) and found a dose-dependent decrease in cathepsin B expression at both the mRNA and protein levels and reduced cell invasiveness [95]. However, under other conditions, cathepsin B might mediate cancer cell apoptosis. PS-341 (bortezomib) is a proteosome inhibitor with broad antitumor activity. It induces the redistribution of lysosomal cathepsin B to the cytosol and activates downstream caspase-2 to induce mitochondrial depolarization and apoptosis in pancreatic cancer cells [96]. The function of AGR2 in PDAC is the same as that in gastric cancer. Procathepsin D secretion is strongly inhibited in AGR2-silenced FA6 cells [97]. S100P belongs to the S100 calcium-binding protein family and can increase cathepsin D expression to activate pancreatic cancer cell invasion [98].

Of course, inhibitors have also been used and detected in pancreatic cancer. For example, VBY-825 can reversibly inhibit cathepsins B, L and S to decrease tumor growth in a mouse pancreatic islet cancer model [99].

## LIVER CANCER

Compared with the previously described digestive cancers, fewer studies have investigated cathepsins in liver cancer. Some studies have shown that the serum levels of cathepsins B and L are increased in patients with liver cancer [100]. However, unlike the high level of cathepsins in other digestive cancers, Lingyu Qin et al. showed that data from the Oncomine database revealed a significantly lower level of cathepsin B mRNA in hepatocellular carcinoma (HCC) than in the corresponding normal tissues. However, cathepsin B is significantly associated with survival and tumor grade. Patients with lower cathepsin B expression have worse overall survival, and cathepsin B expression might be an independent prognostic marker for patients with HCC [101].

Decades ago, cathepsins B and D were implicated in HCC cell apoptosis, and these cathepsins exert protective effects in this cancer. The higher expression of cathepsins B and D is associated with a higher rate of apoptosis. Both the cathepsin B-specific inhibitor CA-074 Me and the cathepsin D inhibitor pepstatin A can markedly decrease apoptosis. An *in vitro* study conducted in HepG2 cells demonstrated that cathepsin B cleaves B cell lymphoma-2 (Bcl-2), an inhibitor of apoptosis, and reduces the Bcl-2/Bax ratio to speed up cell death [102]. Cathepsins in liver cancer might undergo contrasting regulation through an as-yet-unknown mechanism, but cathepsin B- or D-specific agents might be developed as treatments for hepatocellular carcinoma.

As shown in the study by Pinlaor et al. [103], cathepsin F is expressed by O. *viverrini*, and its gene and protein levels are increased in the human liver fluke, *O. viverrini*, which might contribute to hepatobiliary abnormalities, such as cholangiocarcinoma.

## CONCLUSIONS

Cathepsins participate in a wide range of organism activities based on their hydrolysis effect. In digestive cancers, the expression of cathepsins is up-regulated by tumor-promoting factors, such as C-myc, K-ras, AGR2, MAPK, p38, and the Hh signaling pathways. In digestive cancer cells, activated cathepsins hydrolyze growth factors, such as EGF, VEGF, and TGFβ, to induce their maturation and promote cancer cell proliferation. In contrast, cathepsins degrade extracellular matrix proteins to accelerate cancer cell invasion and metastasis. In most digestive cancers, cathepsins promote cancer development but might have opposite effects in liver cancer. Although the roles of cathepsins in tumorigenesis require further investigation, these enzymes might be potential targets in the discovery of drugs that can be used for the treatment of digestive cancers.

## Abbreviations

ECM: extracellular matrix
GAGs: glycosaminoglycans
APC: adenomatous polyposis coli
TP53: tumor protein 53
TGFβ: transforming growth factor-β
Bcl-2: B cell lymphoma-2
AGR2: anterior gradient 2
hTERT: human telomerase reverse transcriptase
RPLP0: ribosomal protein P0
PDAC: pancreatic ductal adenocarcinoma
Hh: Hedgehog
HCC: hepatocellular carcinoma

## References

[1] Turk V, Stoka V, Vasiljeva O, Renko M, Sun T, Turk B, Turk D (2012). Cysteine cathepsins: from structure, function and regulation to new frontiers. Biochim Biophys Acta.

[2] Reinheckel T, Deussing J, Roth W, Peters C (2001). Towards specific functions of lysosomal cysteine peptidases: phenotypes of mice deficient for cathepsin B or cathepsin L. Biol Chem.

[3] Saftig P, Hunziker E, Wehmeyer O, Jones S, Boyde A, Rommerskirch W, Moritz JD, Schu P, von Figura K (1998). Impaired osteoclastic bone resorption leads to osteopetrosis in cathepsin-K-deficient mice. Proc Natl Acad Sci U S A.

[4] Shi GP, Villadangos JA, Dranoff G, Small C, Gu L, Haley KJ, Riese R, Ploegh HL, Chapman HA (1999). Cathepsin S required for normal MHC class II peptide loading and germinal center development. Immunity.

[5] Turk V, Turk B, Guncar G, Turk D, Kos J (2002). Lysosomal cathepsins: structure, role in antigen processing and presentation, and cancer. Adv Enzyme Regul.

[6] Turk B, Turk D, Turk V (2000). Lysosomal cysteine proteases: more than scavengers. Biochim Biophys Acta (BBA) - Protein Struct Mol Enzym.

[7] Vasiljeva O, Reinheckel T, Peters C, Turk D, Turk V, Turk B (2007). Emerging roles of cysteine cathepsins in disease and their potential as drug targets. Curr Pharm Des.

[8] Sloane BF, Dunn JR, Honn KV (1981). Lysosomal cathepsin B: correlation with metastatic potential. Science.

[9] Hamalisto S, Jaattela M (2016). Lysosomes in cancer-living on the edge (of the cell). Curr Opin Cell Biol.

[10] Mason SD, Joyce JA (2011). Proteolytic networks in cancer. Trends Cell Biol.

[11] Velasco G, Ferrando AA, Puente XS, Sanchez LM, Lopez-Otin C (1994). Human cathepsin O. Molecular cloning from a breast carcinoma, production of the active enzyme in Escherichia coli, and expression analysis in human tissues. J Biol Chem.

[12] Hirano T, Takeuchi S (1994). Serum cathepsin-B levels and urinary-excretion of cathepsin-B in the patients with colorectal-cancer - possible indicators for tumor malignancy. Int J Oncol.

[13] Kos J, Šmid A, Krašovec M, Svetic B, Lenarčič B, Vrhovec I, Škrk J, Turk V (1995). Lysosomal proteases cathepsins D, B, H, L and their inhibitors stefins A and B in head and neck cancer. Biol Chem Hoppe Seyler.

[14] Khan A, Krishna M, Baker SP, Malhothra R, Banner BF (1998). Cathepsin B expression and its correlation with tumor-associated laminin and tumor progression in gastric cancer. Arch Pathol Lab Med.

[15] Fernandez PL, Farre X, Nadal A, Fernandez E, Peiro N, Sloane BF, Shi GP, Chapman HA, Campo E, Cardesa A (2001). Expression of cathepsins B and S in the progression of prostate carcinoma. Int J Cancer.

[16] Talieri M, Papadopoulou S, Scorilas A, Xynopoulos D, Arnogianaki N, Plataniotis G, Yotis J, Agnanti N (2004). Cathepsin B and cathepsin D expression in the progression of colorectal adenoma to carcinoma. Cancer Lett.

[17] Campo E, Munoz J, Miquel R, Palacin A, Cardesa A, Sloane BF, Emmert-Buck MR (1994). Cathepsin B expression in colorectal carcinomas correlates with tumor progression and shortened patient survival. Am J Pathol.

[18] Lah TT, Cercek M, Blejec A, Kos J, Gorodetsky E, Somers R, Daskal I, Cathepsin B (2000). a prognostic indicator in lymph node-negative breast carcinoma patients: comparison with cathepsin D, cathepsin L, and other clinical indicators. Clin Cancer Res.

[19] Scorilas A, Fotiou S, Tsiambas E, Yotis J, Kotsiandri F, Sameni M, Sloane BF, Talieri M (2002). Determination of cathepsin B expression may offer additional prognostic information for ovarian cancer patients. Biol Chem.

[20] Palermo C, Joyce JA (2008). Cysteine cathepsin proteases as pharmacological targets in cancer. Trends Pharmacol Sci.

[21] Roshy S, Sloane BF, Moin K (2003). Pericellular cathepsin B and malignant progression. Cancer Metastasis Rev.

[22] Saftig P, Klumperman J (2009). Lysosome biogenesis and lysosomal membrane proteins: trafficking meets function. Nat Rev Mol Cell Biol.

[23] Hasilik A, Wrocklage C, Schröder B (2008). Intracellular trafficking of lysosomal proteins and lysosomes. Int J Clin Pharm Ther.

[24] Schroder BA, Wrocklage C, Hasilik A, Saftig P (2010). The proteome of lysosomes. Proteomics.

[25] Coutinho MF, Prata MJ, Alves S (2012). A shortcut to the lysosome: the mannose-6-phosphate-independent pathway. Mol Genet Metab.

[26] Bromme D, Nallaseth FS, Turk B (2004). Production and activation of recombinant papain-like cysteine proteases. Methods.

[27] Rowan AD, Mason P, Mach L, Mort JS (1992). Rat procathepsin B. Proteolytic processing to the mature form in vitro. J Biol Chem.

[28] Mach L, Mort JS, Glossl J (1994). Maturation of human procathepsin B. Proenzyme activation and proteolytic processing of the precursor to the mature proteinase, in vitro, are primarily unimolecular processes. J Biol Chem.

[29] Ménard R, Carmona E, Takebe S, É Dufour, Plouffe C, Mason P, Mort JS (1998). Autocatalytic processing of recombinant human procathepsin L contribution of both intermolecular and unimolecular events in the processing of procathepsin L in vitro. J Biol Chem.

[30] Jerala R, Zerovnik E, Kidric J, Turk V (1998). pH-induced conformational transitions of the propeptide of human cathepsin LA role for a molten globule state in zymogen activation. J Biol Chem.

[31] Fonović M, Turk B (2014). Cysteine cathepsins and extracellular matrix degradation. Biochim Biophys Acta.

[32] Caglič D, Pungerčar JR, Pejler G, Turk V, Turk B (2007). Glycosaminoglycans facilitate procathepsin B activation through disruption of propeptide-mature enzyme interactions. J Biol Chem.

[33] Vasiljeva O, Dolinar M, Pungerčar JR, Turk V, Turk B (2005). Recombinant human procathepsin S is capable of autocatalytic processing at neutral pH in the presence of glycosaminoglycans. FEBS Lett.

[34] Nissler K, Kreusch S, Rommerskirch W, Strubel W, Weber E, Wiederanders B (1998). Sorting of non-glycosylated human procathepsin S in mammalian cells. Biol Chem.

[35] Nishimura Y, Kawabata T, Furuno K, Kato K (1989). Evidence that aspartic proteinase is involved in the proteolytic processing event of procathepsin L in lysosomes. Arch Biochem Biophys.

[36] Duncan EM, Muratore-Schroeder TL, Cook RG, Garcia BA, Shabanowitz J, Hunt DF, Allis CD (2008). Cathepsin L proteolytically processes histone H3 during mouse embryonic stem cell differentiation. Cell.

[37] Joyce JA, Hanahan D (2004). Multiple roles for cysteine cathepsins in cancer. Cell Cycle.

[38] Joyce JA, Baruch A, Chehade K, Meyer-Morse N, Giraudo E, Tsai FY, Greenbaum DC, Hager JH, Bogyo M, Hanahan D (2004). Cathepsin cysteine proteases are effectors of invasive growth and angiogenesis during multistage tumorigenesis. Cancer Cell.

[39] Tobin DJ, Foitzik K, Reinheckel T, Mecklenburg L, Botchkarev VA, Peters C, Paus R (2002). The lysosomal protease cathepsin L is an important regulator of keratinocyte and melanocyte differentiation during hair follicle morphogenesis and cycling. Am J Pathol.

[40] Berdowska I (2004). Cysteine proteases as disease markers. Clinica Chimica Acta.

[41] Akkari L, Gocheva V, Quick ML, Kester JC, Spencer AK, Garfall AL, Bowman RL, Joyce JA (2016). Combined deletion of cathepsin protease family members reveals compensatory mechanisms in cancer. Genes Dev.

[42] Palesch D, Wagner J, Meid A, Molenda N, Sienczyk M, Burkhardt J, Munch J, Prokop L, Stevanovic S, Westhoff MA, Halatsch ME, Wirtz CR, Zimecki M (2016). Cathepsin G-mediated proteolytic degradation of MHC class I molecules to facilitate immune detection of human glioblastoma cells. Cancer Immunol Immunother.

[43] Lazner F, Gowen M, Kola I (1999). An animal model for pycnodysostosis: the role of cathepsin K in bone remodelling. Mol Med Today.

[44] Mort JS, Buttle DJ, Cathepsin B (1997). Int J Biochem Cell Biol.

[45] Reiser J, Adair B, Reinheckel T (2010). Specialized roles for cysteine cathepsins in health and disease. J Clin Invest.

[46] Urbanelli L, Emiliani C, Massini C, Persichetti E, Orlacchio A, Pelicci G, Sorbi S, Hasilik A, Bernardi G, Orlacchio A (2008). Cathepsin D expression is decreased in Alzheimer's disease fibroblasts. Neurobiol Aging.

[47] Lecaille F, Kaleta J, Brömme D (2002). Human and parasitic papain-like cysteine proteases: their role in physiology and pathology and recent developments in inhibitor design. Chem Rev.

[48] Otto HH, Schirmeister T (1997). Cysteine proteases and their inhibitors. Chem Rev.

[49] McGrath ME (1999). The lysosomal cysteine proteases. Annu Rev Biophys Biomol Struct.

[50] Greenspan PD, Clark KL, Tommasi RA, Cowen SD, McQuire LW, Farley DL, van Duzer JH, Goldberg RL, Zhou H, Du Z (2001). Identification of dipeptidyl nitriles as potent and selective inhibitors of cathepsin B through structure-based drug design. J Med Chem.

[51] Cywin CL, Firestone RA, McNeil DW, Grygon CA, Crane KM, White DM, Kinkade PR, Hopkins JL, Davidson W, Labadia ME, Wildeson J, Morelock MM, Peterson JD (2003). The design of potent hydrazones and disulfides as cathepsin S inhibitors. Bioorg Med Chem.

[52] Duffy KJ, Ridgers LH, DesJarlais RL, Tomaszek TA, Bossard MJ, Thompson SK, Keenan RM, Veber DF (1999). Design and synthesis of diaminopyrrolidinone inhibitors of human osteoclast cathepsin K. Bioorg Med Chem Lett.

[53] Zhou NE, Guo D, Kaleta J, Purisima E, Menard R, Micetich RG, Singh R (2002). Design and synthesis of 6-substituted amino-4-oxa-1-azabicyclo [3, 2, 0] heptan-7-one derivatives as cysteine proteases inhibitors. Bioorg Med Chem Lett.

[54] Pawelczap M, Nowak K, Kafarski P (1998). Synthesis of phosphono dipeptides, inhibitors of cathepsin C. Phosphorus Sulfur Silicon Relat Elem.

[55] Hara T, Ooi A, Kobayashi M, Mai M, Yanagihara K, Nakanishi I (1998). Amplification of c-myc, K-sam, and c-met in gastric cancers: detection by fluorescence in situ hybridization. Lab Invest.

[56] He TC, Sparks AB, Rago C, Hermeking H, Zawel L, LT Da Costa, Morin PJ, Vogelstein B, Kinzler KW (1998). Identification of c-MYC as a target of the APC pathway. Science.

[57] Levine AJ (1997). p53, the cellular gatekeeper for growth and division. Cell.

[58] Enzinger PC, Mayer RJ (2003). Esophageal cancer. N Engl J Med.

[59] Fisher OM, Levert-Mignon AJ, Lord SJ, Botelho NK, Freeman AK, Thomas ML, Falkenback D, Wettstein A, Whiteman DC, Bobryshev YV (2015). High expression of Cathepsin E in tissues but not blood of patients with Barrett's esophagus and adenocarcinoma. Ann Surg Oncol.

[60] Ma W, Ma L, Zhe H, Bao C, Wang N, Yang S, Wang K, Cao F, Cheng Y, Cheng Y (2014). Detection of esophageal squamous cell carcinoma by cathepsin B activity in nude mice. PLoS One.

[61] McCabe ML, Dlamini Z (2005). The molecular mechanisms of oesophageal cancer. Int Immunopharmacol.

[62] Habibollahi P, Figueiredo JL, Heidari P, Dulak AM, Imamura Y, Bass AJ, Ogino S, Chan AT, Mahmood U (2012). Optical imaging with a cathepsin B activated probe for the enhanced detection of esophageal adenocarcinoma by dual channel fluorescent upper GI endoscopy. Theranostics.

[63] Andl CD, McCowan KM, Allison GL, Rustgi AK (2010). Cathepsin B is the driving force of esophageal cell invasion in a fibroblast-dependent manner. Neoplasia.

[64] Szumilo J, Burdan F, Zinkiewicz K, Dudka J, Klepacz R, Dabrowski A, Korobowicz E (2009). Expression of syndecan-1 and cathepsins D and K in advanced esophageal squamous cell carcinoma. Folia Histochem Cytobiol.

[65] Ikeguchi M, Sakatani T, Ueta T, Fukuda K, Oka S, Hisamitsu K, Yamaguchi K, Tsujitani S, Kaibara N (2002). Correlation between cathepsin D expression and p53 protein nuclear accumulation in oesophageal squamous cell carcinoma. J Clin Pathol.

[66] Xu L, Peng S, Zhang N, Liu R, Huang Q, Li X, Wang J (2016). Expression status of cathepsin B may as a prognostic marker for human gastric carcinoma. Int J Clin Exp Pathol.

[67] Ikeguchi M, Fukuda K, Oka S, Hisamitsu K, Katano K, Tsujitani S, Kaibara N (2001). Micro-lymph node metastasis and its correlation with cathepsin D expression in early gastric cancer. J Surg Oncol.

[68] He B, Xiao YF, Tang B, Wu YY, Hu CJ, Xie R, Yang X, Yu ST, Dong H, Zhao XY, Li JL, Yang SM (2016). hTERT mediates gastric cancer metastasis partially through the indirect targeting of ITGB1 by microRNA-29a. Sci Rep.

[69] Zhang J, Jin Y, Xu S, Zheng J, Zhang Q, Wang Y, Chen J, Huang Y, He X, Zhao Z (2016). AGR2 is associated with gastric cancer progression and poor survival. Oncol Lett.

[70] Allgayer H, Heiss MM, Schildberg FW (1997). Prognostic factors in gastric cancer. Br J Surg.

[71] Matsuo K, Kobayashi I, Tsukuba T, Kiyoshima T, Ishibashi Y, Miyoshi A, Yamamoto K, Sakai H (1996). Immunohistochemical localization of cathepsins D and E in human gastric cancer: a possible correlation with local invasive and metastatic activities of carcinoma cells. Hum Pathol.

[72] Ebert MP, Kruger S, Fogeron ML, Lamer S, Chen J, Pross M, Schulz HU, Lage H, Heim S, Roessner A, Malfertheiner P, Rocken C (2005). Overexpression of cathepsin B in gastric cancer identified by proteome analysis. Proteomics.

[73] Atwa HA, Arafa SA (2016). Significance of TGF-β1 and Catheps in E expression in gastric adenocarcinoma and precancerouslesions. J Am Sci.

[74] Marin HE, Peraza MA, Billin AN, Willson TM, Ward JM, Kennett MJ, Gonzalez FJ, Peters JM (2006). Ligand activation of peroxisome proliferator-activated receptor beta inhibits colon carcinogenesis. Cancer Res.

[75] Konno-Shimizu M, Yamamichi N, Inada K, Kageyama-Yahara N, Shiogama K, Takahashi Y, Asada-Hirayama I, Yamamichi-Nishina M, Nakayama C, Ono S (2013). Cathepsin E is a marker of gastric differentiation and signet-ring cell carcinoma of stomach: a novel suggestion on gastric tumorigenesis. PLoS One.

[76] Ren G, Tian Q, An Y, Feng B, Lu Y, Liang J, Li K, Shang Y, Nie Y, Wang X (2012). Coronin 3 promotes gastric cancer metastasis via the up-regulation of MMP-9 and cathepsin K. Mol Cancer.

[77] Yu S, Yu Y, Zhang W, Yuan W, Zhao N, Li Q, Cui Y, Wang Y, Li W, Sun Y, Liu T (2016). FOXO3a promotes gastric cancer cell migration and invasion through the induction of cathepsin L. Oncotarget.

[78] Dohchin A, Suzuki JI, Seki H, Masutani M, Shiroto H, Kawakami Y (2000). Immunostained cathepsins B and L correlate with depth of invasion and different metastatic pathways in early stage gastric carcinoma. Cancer.

[79] Liu WL, Liu D, Cheng K, Liu YJ, Xing S, Chi PD, Liu XH, Xue N, Lai YZ, Guo L, Zhang G (2016). Evaluating the diagnostic and prognostic value of circulating cathepsin S in gastric cancer. Oncotarget.

[80] Krueger S, Kalinski T, Hundertmark T, Wex T, Kuster D, Peitz U, Ebert M, Nagler DK, Kellner U, Malfertheiner P, Naumann M, Rocken C, Roessner A (2005). Up-regulation of cathepsin X in Helicobacter pylori gastritis and gastric cancer. J Pathol.

[81] Teller A, Jechorek D, Hartig R, Adolf D, Reissig K, Roessner A, Franke S (2015). Dysregulation of apoptotic signaling pathways by interaction of RPLP0 and cathepsin X/Z in gastric cancer. Pathol Res Pract.

[82] Choi EH, Kim JT, Kim JH, Kim SY, Song EY, Kim JW, Kim SY, Yeom YI, Kim IH, Lee HG (2009). Upregulation of the cysteine protease inhibitor, cystatin SN, contributes to cell proliferation and cathepsin inhibition in gastric cancer. Clin Chim Acta.

[83] Shao LH, Liu SP, Hou JX, Zhang YH, Peng CW, Zhong YJ, Liu X, Liu XL, Hong YP, Firestone RA (2012). Cathepsin B cleavable novel prodrug Ac-Phe-Lys-PABC-ADM enhances efficacy at reduced toxicity in treating gastric cancer peritoneal carcinomatosis. Cancer.

[84] Hazen LG, Bleeker FE, Lauritzen B, Bahns S, Song J, Jonker A, Van Driel BE, Lyon H, Hansen U, Kohler A, Van Noorden CJ (2000). Comparative localization of cathepsin B protein and activity in colorectal cancer. J Histochem Cytochem.

[85] Chan AT, Baba Y, Shima K, Nosho K, Chung DC, Hung KE, Mahmood U, Madden K, Poss K, Ranieri A, Shue D, Kucherlapati R, Fuchs CS (2010). Cathepsin B expression and survival in colon cancer: implications for molecular detection of neoplasia. Cancer Epidemiol Biomark Prev.

[86] Cavallo-Medved D, Dosescu J, Linebaugh BE, Sameni M, Rudy D, Sloane BF (2003). Mutant K-ras regulates cathepsin B localization on the surface of human colorectal carcinoma cells. Neoplasia.

[87] Jechorek D, Votapek J, Meyer F, Kandulski A, Roessner A, Franke S (2014). Characterization of cathepsin X in colorectal cancer development and progression. Pathol Res Pract.

[88] Li D, Xie K, Wolff R, Abbruzzese JL (2004). Pancreatic cancer. Lancet.

[89] Niedergethmann M, Hildenbrand R, Wolf G, Verbeke CS, Richter A, Post S (2000). Angiogenesis and cathepsin expression are prognostic factors in pancreatic adenocarcinoma after curative resection. Int J Pancreatol.

[90] Singh N, Das P, Gupta S, Sachdev V, Srivasatava S, Datta Gupta S, Pandey RM, Sahni P, Chauhan SS, Saraya A (2014). Plasma cathepsin L: a prognostic marker for pancreatic cancer. World J Gastroenterol.

[91] Brindle NR, Joyce JA, Rostker F, Lawlor ER, Swigart-Brown L, Evan G, Hanahan D, Shchors K (2015). Deficiency for the cysteine protease cathepsin L impairs Myc-induced tumorigenesis in a mouse model of pancreatic neuroendocrine cancer. PLoS One.

[92] Cruz-Monserrate Z, Abd-Elgaliel WR, Grote T, Deng D, Ji B, Arumugam T, Wang H, Tung CH, Logsdon CD (2012). Detection of pancreatic cancer tumours and precursor lesions by cathepsin E activity in mouse models. Gut.

[93] Li H, Li Y, Cui L, Wang B, Cui W, Li M, Cheng Y (2014). Monitoring pancreatic carcinogenesis by the molecular imaging of cathepsin E in vivo using confocal laser endomicroscopy. PLoS One.

[94] Uno K, Azuma T, Nakajima M, Yasuda K, Hayakumo T, Mukai H, Sakai T, Kawai K (2000). Clinical significance of cathepsin E in pancreatic juice in the diagnosis of pancreatic ductal adenocarcinoma. J Gastroenterol Hepatol.

[95] Hwang JH, Lee SH, Lee KH, Lee KY, Kim H, Ryu JK, Yoon YB, Kim YT (2009). Cathepsin B is a target of Hedgehog signaling in pancreatic cancer. Cancer Lett.

[96] Yeung BH, Huang DC, Sinicrope FA (2006). PS-341 (bortezomib) induces lysosomal cathepsin B release and a caspase-2-dependent mitochondrial permeabilization and apoptosis in human pancreatic cancer cells. J Biol Chem.

[97] Dumartin L, Whiteman HJ, Weeks ME, Hariharan D, Dmitrovic B, Iacobuzio-Donahue CA, Brentnall TA, Bronner MP, Feakins RM, Timms JF (2011). AGR2 is a novel surface antigen that promotes the dissemination of pancreatic cancer cells through regulation of cathepsins B and D. Cancer Res.

[98] Whiteman HJ, Weeks ME, Dowen SE, Barry S, Timms JF, Lemoine NR, Crnogorac-Jurcevic T (2007). The role of S100P in the invasion of pancreatic cancer cells is mediated through cytoskeletal changes and regulation of cathepsin D. Cancer Res.

[99] Elie BT, Gocheva V, Shree T, Dalrymple SA, Holsinger LJ, Joyce JA (2010). Identification and pre-clinical testing of a reversible cathepsin protease inhibitor reveals anti-tumor efficacy in a pancreatic cancer model. Biochimie.

[100] Leto G, Tumminello FM, Pizzolanti G, Montalto G, Soresi M, Gebbia N (2009). Lysosomal cathepsins B and L and Stef in A blood levels in patients with hepatocellular carcinoma and/or liver cirrhosis: potential clinical implications. Oncology.

[101] Qin L, Chen J, Wang J, Ye J, Tan H, Xu L (2016). Expression of cathepsin B in human hepatocellular carcinoma and its prognostic significance. Int J Clin Exp Pathol.

[102] Droga-Mazovec G, Bojič L, Petelin A, Ivanova S, Repnik U, Salvesen GS, Stoka V, Turk V, Turk B (2008). Cysteine cathepsins trigger caspase-dependent cell death through cleavage of bid and antiapoptotic Bcl-2 homologues. J Biol Chem.

[103] Pinlaor P, Kaewpitoon N, Laha T, Sripa B, Kaewkes S, Morales ME, Mann VH, Parriott SK, Suttiprapa S, Robinson MW (2009). Cathepsin F cysteine protease of the human liver fluke, Opisthorchis viverrini. PLoS Negl Trop Dis.

